# Quantitative evaluation of the vertical mobility of the first tarsometatarsal joint during stance phase of gait

**DOI:** 10.1038/s41598-022-13425-5

**Published:** 2022-06-02

**Authors:** Noriaki Maeda, Yasunari Ikuta, Tsubasa Tashiro, Satoshi Arima, Masanori Morikawa, Kazuki Kaneda, Honoka Ishihara, Andreas Brand, Tomoyuki Nakasa, Nobuo Adachi, Yukio Urabe

**Affiliations:** 1 Department of Sports Rehabilitation, Graduate School of Biomedical and Health Sciences, Hiroshima University, 1-2-3 Kasumi, Minami-ku, Hiroshima, 734-8553 Japan; 2 Department of Orthopaedic Surgery, Graduate School of Biomedical and Health Sciences, Hiroshima University, 1-2-3 Kasumi, Minami-ku, Hiroshima, 734-8553 Japan; 3 Sports Medical Center, Hiroshima University Hospital, Hiroshima, Japan; 4 Department of Preventive Gerontology, Center for Gerontology and Social Science, National Center for Geriatrics and Gerontology, Aichi, Japan; 5 Institute for Biomechanics, BG Unfallklinik Murnau, Murnau, Germany; 6 Institute for Biomechanics, Paracelsus Medical Private University Salzburg, Salzburg, Austria; 7 Medical Center for Translational and Clinical Research, Hiroshima University Hospital, Hiroshima, Japan

**Keywords:** Bone, Bone quality and biomechanics

## Abstract

We determined how the in vivo mobility of the first tarsometatarsal (TMT) joint can be quantified during gait. Twenty-five healthy participants (12 females) with no history of foot disorders were included. Non-invasive ultrasound (US) with a three-dimensional motion analysis (MA) system was used to evaluate the kinematic characteristics of first TMT joint during stance phase of gait. US probe was positioned longitudinally above the first TMT joint and adjusted to its proximal dorsal prominence. Gait analysis was conducted by the MA system starting with the activation of B-mode US video at 80 frames per second and 60-mm depth for simultaneous capture. During stance phase, the first metatarsal was translated dorsally with respect to the medial cuneiform, returning to a neutral level at toe-off in all subjects. During middle stance phase, the medial cuneiform was stable in males but displaced in the plantar direction in females and was the primary contributor to the differences in sagittal mobility observed between groups. Quantitatively measuring sagittal mobility of the first TMT joint could be useful for the early detection of foot abnormalities. The dynamic characteristics of the medial cuneiform during gait in healthy females may be associated with a high prevalence of hallux valgus.

## Introduction

Hallux valgus (HV) is a common foot deformity, with a prevalence of 23% in adults aged 18–65 years and 35.7% in adults aged > 65 years^1^. Several intrinsic factors are associated with the pathogenesis of HV, including female sex, age, metatarsal morphology, and first-ray hypermobility^2^. Hypermobility between the first metatarsal and medial cuneiform is one cause of HV, due to the laxity of the Lisfranc joint ligament and decreased plantar arch height^3^. Studies have reported that instability of the first TMT joint was identified in 94%–96% of patients with HV^4,5^. Hypermobility of the first TMT joint is correlated with the severity of HV and is compounded by repetitive downward movements of the metatarsals during weight bearing^6,7^. Biomechanically, excessive first metatarsal extension during the stance phase of gait leads to ligamentous laxity in the metatarsals and subtalar joints, reducing foot stiffness during the terminal stance and adversely affecting push-off mechanics^6^.

However, the hypermobility of isolated sagittal planes of the first TMT joint has not yet been shown as a definitive cause of HV^8^.

The mobility of the first ray and TMT joint in sagittal plane has been evaluated using several methods, such as manual examination^9^, a metal ruler^10^, a modified ankle–foot-orthosis with an adjustable micrometer^11^, lateral weight-bearing radiographs^12^, and three-dimensional computed tomography images with weight-bearing^13^. The accuracy of the manual test for the assessment of first-ray mobility is controversial, as the subjective results of the manual test are not quantifiable^14^. A few studies have reported the mobility of the first TMT joint using original devices^4,10^. However, their quantitative evaluations were limited, as the measurements were obtained manually with the feet immobilized, yielding only static observations. The average standard value of the first TMT joint mobility is 3.6–4.7 mm; however, these values are based on static measurements^10^. The mobility of the first TMT joint has been evaluated in several cadaveric studies^15^. In the study of Coughlin MJ et al., the first TMT joint was simulated in three-dimensional space; however, ligaments and other soft tissues were different from those in vivo, possibly affecting joint mobility. The in vivo dynamic gait assessment was performed using a digital fluoroscopic-based method for the rotational and translational movements of the first metatarsal, medial cuneiform, navicular, and talus during the stance phase of gait^16^. Particularly concerning are the patient’s radiation exposure and unclear images due to a frequency of 25 frames per second. Additionally, the angle of the first TMT joint during gait was evaluated using a three-dimensional MA system^17^; however, detailed observations for joint movements of the foot are limited. Thus, in vivo quantitative evaluation of the first TMT joint during gait has been challenging previously.

This study aimed to establish a method to quantitatively evaluate the mobility of the first TMT joint in vivo during the stance phase. A novel combination of ultrasound (US) imaging and a three-dimensional MA system was applied to assess first TMT joint mobility during gait in healthy participants. Further, HV is more prevalent in females, due to the differences in bone structure, joint laxity, and their habit of wearing tight-fitting shoes^2,18^. Therefore, we investigated the differences in the first TMT joint mobility between females and males. We hypothesize that the mobilities of the first metatarsal and medial cuneiform during the stance phase are greater in females than in males.

## Methods

### Participants

Participants were 25 healthy, recreationally active individuals, including 12 females and 13 males. Recreationally active was defined as having participated in at least one exercise session weekly—but not performing structured exercise training—during the preceding 2 months. Participants were included if they had a foot posture index (FPI-6) score < 6 points, and a score ≥ 6 was used to exclude individuals due to pes planus alignment^19^. Participants with foot and ankle problems such as ligament injuries; plantar fasciitis; bursitis; a history of lower extremity trauma; or neurological conditions affecting balance were also excluded.

**Figure 1:**
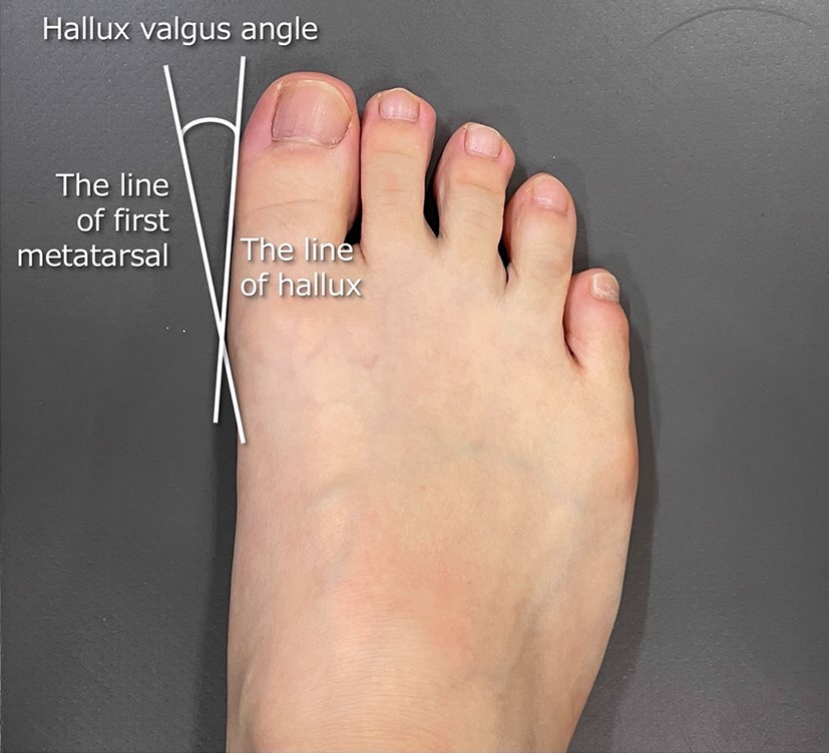
Methodology for determining the hallux valgus angle. This angle was calculated as the angle between the medial aspect of the hallux and first metatarsal on an image taken in the horizontal plane from the height of the participant's knee.

Foot deformities were evaluated using photographs of the participant’s leg (front side) from the knees to feet. The angle between the medial aspect of the hallux and the first metatarsal (HV angle) was measured using these images (Fig. 1)^20^.

This study was approved by the Ethical Committee for Epidemiology of Hiroshima University (Approval Number: E-2187) and conducted following the principles of the Declaration of Helsinki. All participants provided informed consent.

### Experimental procedure

#### Assessment of first TMT joint mobility using B-mode US during gait

**Figure 2:**
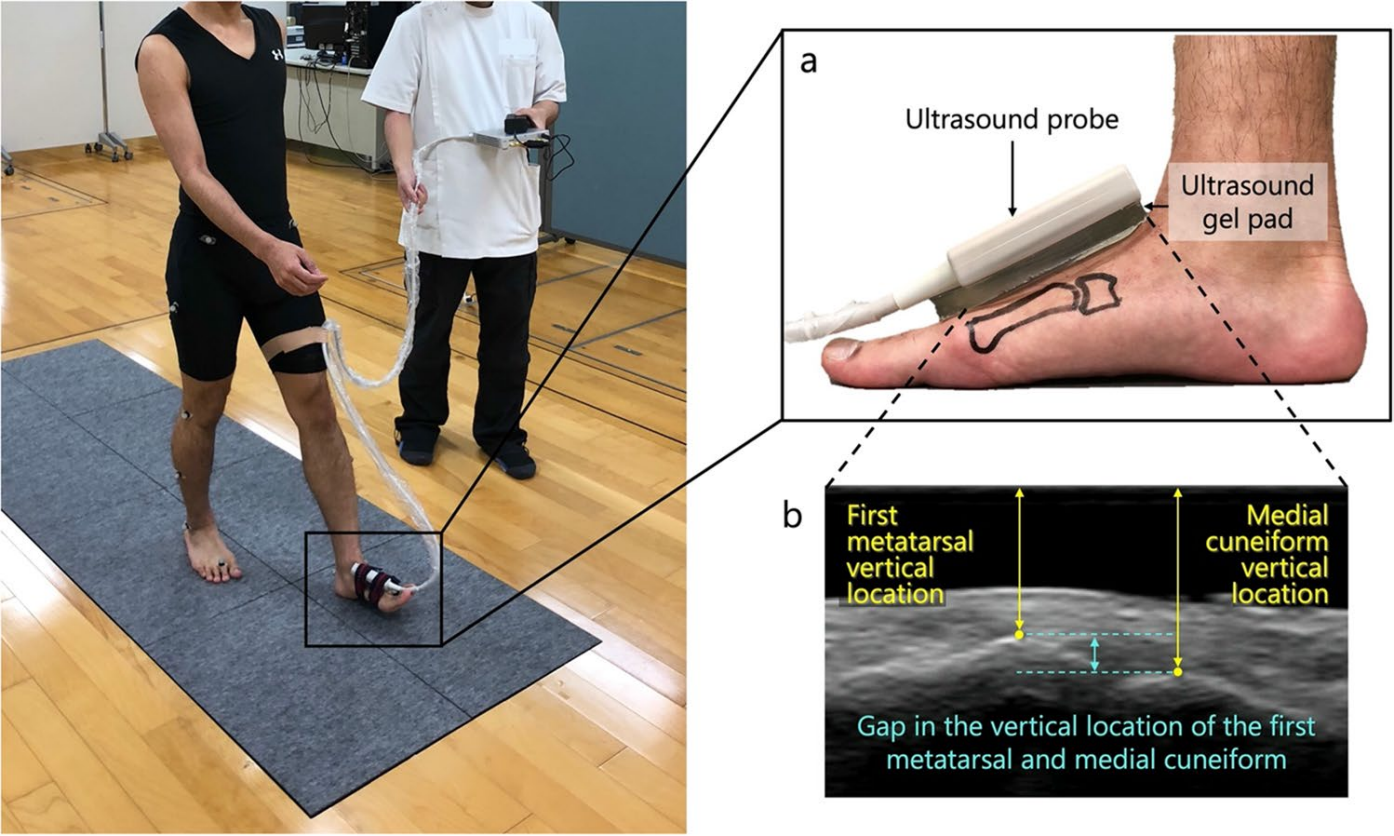
Gait analysis using ultrasound and a three-dimensional motion analysis system. Ultrasound probe was attached at the tarsometatarsal joint on an ultrasound gel pad (**a**), and the first metatarsal and medial cuneiform were within the field of view (**b**). The vertical locations of the first metatarsal and medial cuneiform were defined as the vertical distance from the screen top to the dorsum of each bone.

We used a B-mode US system (Art Us EXT-1H, Telemed, Vilnius, Lithuania) and a US probe (5–11 MHz, 60-mm field of view; Echoblaster, Telemed, Vilnius, Lithuania)^21^ to evaluate the joint motion of the first TMT joint in the sagittal plane during stance phase. The MA system was synchronized with a US system that captured B-mode US video at 80 frames/s and 60 mm depth^22^. To start and stop simultaneous captures, changes in the electrical signal edges produced by the activation/deactivation of the US switch were recognized by the MA system. Captured data samples within both systems were then put in relation according to their predefined frame rates. The US probe settings are shown in Fig. 2. The US videos were visually inspected for each gait test to ensure that the landmarks of the first metatarsal and medial cuneiform did not disappear. If the video was unclear or the probe misaligned, the measurement was performed again. To standardize the placement position of the probe, we marked each participant’s skin and checked for any deviations. Three US video trials were measured for each side.

### Gait analysis

Each participant walked 6 m over eight force plates (OR-6, 1000 Hz: AMTI, USA). Gait was analyzed using the Vicon motion capture analysis system with 16 infrared cameras (100 Hz)^23^. Sixteen reflective markers were placed on the lower body of the participants, and the markers were applied by the same examiner. After acquiring the data in the static standing position, participants walked over the force plates at their natural speed; two practice trials were conducted before measurements were obtained. Data captions took an average of 3 s for all participants. The participants took two steps before walking on the force plates.

### Data analysis

The first of the force plate data were processed using the Plug-in-Gait pipeline for Vicon Nexus ver. 1.8.5 software (Vicon Motion Systems, UK) to detect the gait phase. The phase from heel contact to toe-off of the foot attached to the US probe was defined as one stance phase. One stance phase was calculated for all three trials on each side. Tracker 5.1.5 software (Open Source Physics, https://www.compadre.org) was used to calculate the vertical translation of the metatarsal and medial cuneiform in the US video^24^. Temporal changes in the vertical location of the first TMT joint during one stance phase obtained from the Vicon software were calculated. Vertical locations of the first metatarsal and medial cuneiform were defined as the shortest distance from the top of the video screen to the top of each bone (Fig. 2b); their locations were calculated for all frames. The gap in the vertical location of the medial cuneiform relative to the first metatarsal was analyzed by defining the first frame as the neutral level. One stance phase for each participant was normalized to 100 frames using normalization software. The overall profile was analyzed based on previous activity phases during the gait cycle. Finally, each stride between heel contact and toe-off was analyzed separately for the three stance phases: early (0–33 frames), middle (34–66 frames), and terminal (67–100 frames)^25^.

### Statistical analysis

Outcomes were confirmed using the Shapiro–Wilk test for normality. Student’s *t*-test was used to compare quantitative variables. Intra-rater reliability of vertical movement of the first TMT joint during the three stance phases was assessed with intraclass correlation coefficients (ICC_1,1_). ICC_1,1_ was regarded as excellent if > 0.74, good if 0.60–0.74, fair if 0.40–0.59, and poor if < 0.40. Additionally, the standard error of the measurements (SEM) was calculated for the US data at each stance phase for sexes to evaluate the accuracy of the measurements. A two-way repeated-measures analysis of variance was used to determine the differences in US values between sexes as a between-subject factor and stance phase as a within-participant factor. When interaction effects were detected, post-hoc comparisons were conducted using the Tukey method to test the differences in movement variables of the first metatarsal and medial cuneiform between females and males. Partial η^2^ values were used to measure effect size. The post-hoc observed power based on partial η^2^ was generated using G*Power 3.1 software (Kiel University, Germany). The sample size of 24 feet in the female group and 26 feet in the male group was employed for statistical power analysis. The results of the post-hoc analysis had a large effect size (d = 0.8), an alpha level of p < 0.05, and showed a statistical power of 0.873, indicating adequate power. Statistical significance was set at p < 0.05. Analyses were done using SPSS version 23.0 for Windows (SPSS Inc, Chicago, Illinois, USA).

## Results

The participants’ demographics are shown in Table 1. Mean age (p = 0.007), height (p < 0.001), body weight (p < 0.001), and body mass index (BMI) (p = 0.020) were significantly lower in females than in males. No significant differences in HV angle and FPI-6 scores were found between the two groups.

**Table 1:** General characteristics of healthy subjects.

	Female (n = 12, 24 feet)	Male (n = 13, 26 feet)	p-value
Age (years)	21.3 ± 1.2	24.1 ± 3.0	0.007
Height (cm)	157.1 ± 4.6	171.1 ± 3.8	< 0.001
Body weight (kg)	49.6 ± 3.8	67.1 ± 11.8	< 0.001
Body mass index (kg/m^2^)	20.1 ± 1.8	22.9 ± 3.4	0.020
Hallux valgus angle (°)	11.6 ± 5.0	8.8 ± 3.3	0.122
Foot posture index	2.1 ± 1.2	1.9 ± 2.1	0.570

Data are shown as mean ± standard deviation.

Intra-rater reliability for US vertical movement of the first TMT joint during the stance phase was considered excellent in the location of the metatarsals and medial cuneiform, and good to excellent in the gap of the first metatarsal and medial cuneiform (Table 2).

**Table 2:** Reproducibility of ultrasound vertical movement of the first tarsometatarsal joint during the stance phase of gait.

Variable	Stance phase	Female		Male	
		ICC_1,1_	SEM	ICC_1,1_	SEM
First metatarsal	Early phase	0.993 (0.986–0.997)	0.312	0.953 (0.911–0.977)	0.376
	Middle phase	0.985 (0.971–0.993)	0.453	0.916 (0.846–0.959)	0.492
	Terminal phase	0.980 (0.961–0.991)	0.544	0.924 (0.861–0.963)	0.490
Medial cuneiform	Early phase	0.980 (0.961–0.991)	0.579	0.969 (0.941–0.985)	0.347
	Middle phase	0.977 (0.953–0.989)	0.645	0.949 (0.904–0.975)	0.454
	Terminal phase	0.966 (0.934–0.985)	0.800	0.937 (0.883–0.969)	0.541
Gap in the first metatarsal and medial cuneiform	Early phase	0.651 (0.430–0.820)	0.510	0.879 (0.785–0.939)	0.324
	Middle phase	0.676 (0.465–0.835)	0.560	0.815 (0.683–0.905)	0.405
	Terminal phase	0.64 (0.420–0.815)	0.697	0.740 (0.571–0.862)	0.513

*ICC* intraclass correlations coefficient, *SEM* standard error of the measurements. SEM was calculated using the formula s√1-ICC. Values in parentheses are 95% confidence for ICC and lower and upper limits for SEM.

**Figure 3:**
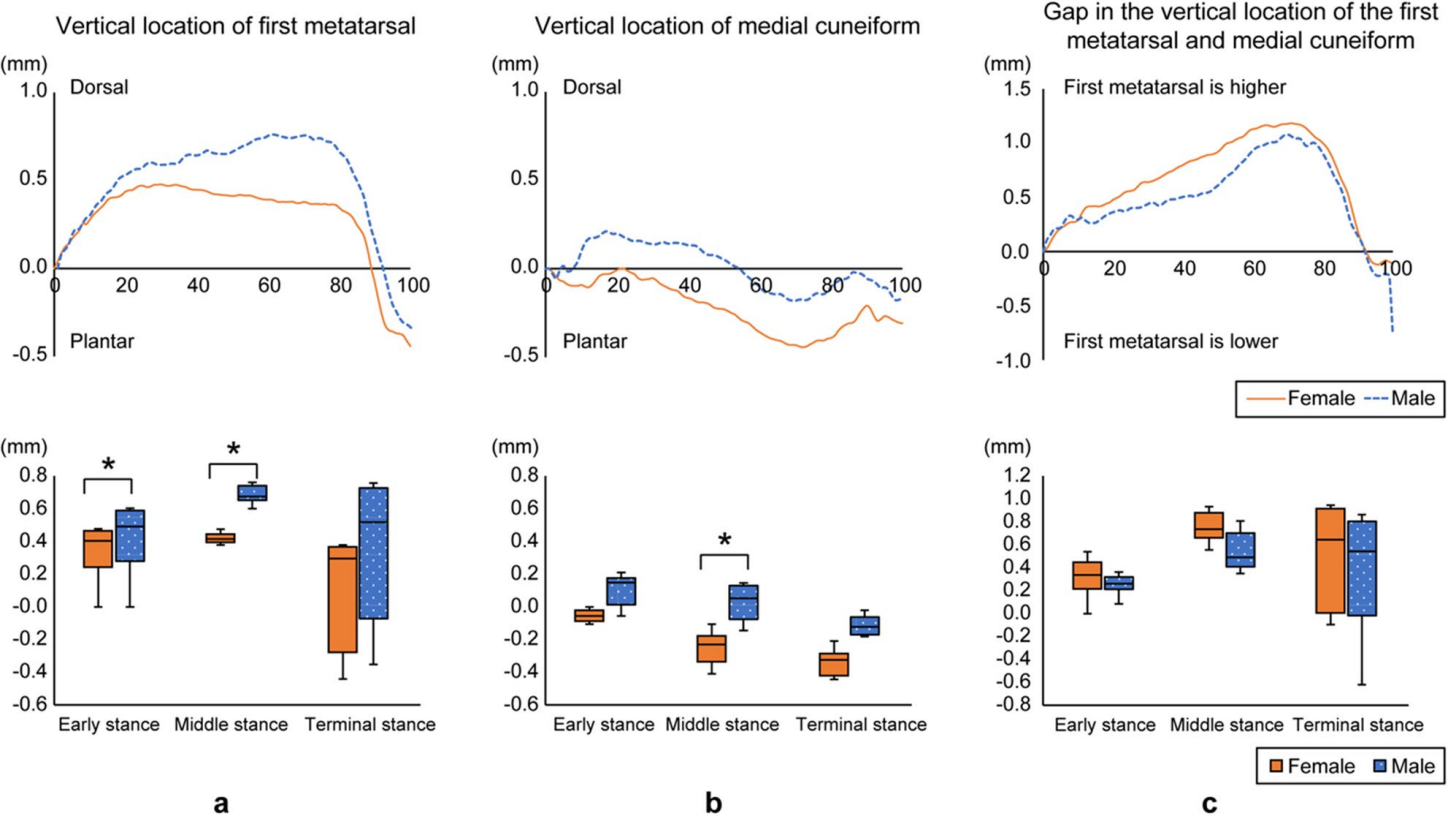
Gender differences in first tarsometatarsal joint movement during one stance phase of gait. Temporal changes in the vertical location of the first metatarsal (**a**), medial cuneiform (**b**), and displacement of the two (**c**) are shown for males and females. Box plots comparing the vertical locations during the early, middle, and terminal stance phases are shown. *p < 0.01. Significant plantar displacement was observed in the vertical location of the first metatarsal in early and middle stance and in the vertical location of the medial cuneiform in middle stance in female compared to male.

The changes in the ankle angle in dorsiflexion-plantar flexion during stance phase with and without the probe were not significantly different (Supplemental Fig. 1). The first metatarsal gradually displaced in the dorsal direction during the early stance phase and maintained the position during the middle stance. In the late phase, the first metatarsal shifted back in the plantar direction. In addition, a more plantar location was observed in females than in males during the early and middle stance phases (Fig. 3a; p = 0.038, p = 0.042, respectively). The position of the medial cuneiform was stable during the entire stance phase, compared with the first metatarsal, which was located more plantar in females than in males during the middle stance phase (Fig. 3b; p = 0.034). The gap of the vertical location of the first metatarsal and medial cuneiform in females tended to be large during the middle stance, compared with males; however, no statistical differences were identified (Fig. 3c).

Participant sex significantly affected the vertical locations of the medial cuneiform during the stance phase (p = 0.025). Gait phase had a significant effect on the first metatarsal, medial cuneiform, and the gap between them (p < 0.001, p = 0.001, p < 0.001, respectively; Table 3).

**Table 3:** Differences in the first metatarsal, medial cuneiform, and gap during stance phase.

	Stance phase												Interaction effect (sex × phase)				Main effect (sex)				Main effect (phase)			
	Early phase				Middle phase				Terminal phase															
	Female	Male	*p*	d	Female	Male	*p*	d	Female	Male	*p*	d	*F*	*p*	η^2^	Observed power	*F*	*p*	η^2^	Observed power	*F*	*p*	η^2^	Observed power
First metatarsal, mm	0.34 ± 0.39	0.42 ± 0.32	0.162	0.230	0.42 ± 0.54	0.69 ± 0.55	0.034	0.500	0.11 ± 0.53	0.35 ± 0.42	0.079	0.509	2.570	0.082	0.051	0.261	2.683	0.108	0.053	0.272	26.467	< 0.001	0.355	0.999
Medial cuneiform, mm	− 0.05 ± 0.31	0.12 ± 0.24	0.038	0.605	− 0.25 ± 0.55	0.02 ± 0.32	0.042	0.609	− 0.34 ± 0.59	− 0.07 ± 0.48	0.081	0.505	0.507	0.568	0.010	0.081	5.364	0.025	0.101	0.530	8.653	0.001	0.153	0.767
Gap in first metatarsal and medial cuneiform, mm	0.34 ± 0.43	0.25 ± 0.28	0.641	0.250	0.75 ± 0.58	0.55 ± 0.57	0.221	− 0.351	0.50 ± 0.66	0.41 ± 0.56	0.598	− 0.150	0.883	0.398	0.018	0.111	0.825	0.368	0.017	0.106	24.756	< 0.001	0.340	0.998

Data are shown as mean ± standard deviation. *d* Cohen’s d, *η*^*2*^ partial eta-squared. Significant effect of participant sex on the vertical location of the medial cuneiform were found. Significant effects of gait phase on the vertical locations of the first metatarsal, medial cuneiform, and the gap between them were found.

## Discussion

This is the first study to quantify the mobility of first TMT joint in sagittal plane during gait using a synchronized US and MA system. The first metatarsal was located dorsally compared to the medial cuneiform during most of the stance phase, returning to neutral level at toe-off. Mobility of the first metatarsal and medial cuneiform differed in females and males, with the medial cuneiform being stable in males and displaced in the plantar direction in females, especially in the middle stance phase. Our US/MA system captured the vertical location and motion of the first metatarsal and medial cuneiform, allowing the assessment of the first TMT joint dynamics during gait.

The TMT joint is vital in maintaining the lateral and longitudinal stability of the midfoot^26^. Midfoot stability is associated with ankle, knee, and hip movement during gait^27^, and hypermobility of the first TMT can lead to collapsing foot arches, which may reduce adaptive abilities during gait. The quantification of the physiological mobility and pathological instability of the first metatarsal and medial cuneiform remains unclear. In this study, we performed a kinematic examination using a novel combination of the US and gait MA during the dynamic rollover phase to further evaluate the detailed biomechanical characteristics of first metatarsal and medial cuneiform during the stance phase.

Significant differences in the movements of the first metatarsal and medial cuneiform during the stance phase were observed between females and males. A study reported that females had more joint mobility of foot and laxity of ligaments supporting the foot arch than males^28^. The prevalence of HV is greater in females and may be related to differences in footwear, osseous anatomy, and ligamentous laxity^2^. Recently, the three-dimensional mobility of first ray, including the talonavicular, cuneonavicular, and first TMT joints, was evaluated using weight-bearing computed tomography images^13^. The first metatarsal showed a mean dorsiflexion angle of 2.0° ± 1.3°, mean inversion angle of 2.6° ± 1.4°, and mean adduction (relative to the medial cuneiform) angle of 1.1° ± 0.7° under loading in middle-aged, healthy volunteers^13^. These mobility values—determined with patients in static standing postures—were significantly larger in HV patients than in individuals with normal feet. This study evaluated the mobility of the first TMT joint in dynamic postures and indicated that the medial cuneiform is displaced in the plantar direction during the middle stance phase in healthy females. This finding suggests more laxity of the naviculocuneiform or intercuneiform 1–2 joints. A cadaveric study demonstrated that the naviculocuneiform, first TMT, and talonavicular joints contributed an average of 50%, 41%, and 9%, respectively, to the total first-ray sagittal plane range of motion^29^. The three-dimensional analysis using weight-bearing computed tomography images revealed greater mobility of the intercuneiform 1–2 joints in individuals with HV than in those with normal feet. A significant increase in dorsiflexion and inversion displacement of the intermediate cuneiform relative to the medial cuneiform has been reported^30^. Plantar displacement of the medial cuneiform in the stance phase might be induced by the hypermobility of the naviculocuneiform, intercuneiform 1–2 joint. These dynamics can lead to subsequent hypermobility of the first TMT joint, and long-term progression of HV and its higher prevalence among females. Thus, future studies investigating the relationship between the first-ray mobility and onset of HV in healthy participants are warranted. Our results can help improve the prognoses and quality of treatment for patients with foot deformities, including HV. It may also aid in developing a new evaluation method for the early detection of abnormalities in the first TMT joint or improving the progression of early-stage foot deformities.

This study has several limitations. First, the participants in this study were younger than the general age group in which foot deformities are observed. Second, our US measurements only observed the vertical mobility of the first TMT joint during gait. Future research should address the multi-directional analysis of the foot joint, and technological progress is essential to resolve this issue. Finally, the mobility of the naviculocuneiform joint could not be evaluated, as probe positioning would interfere with ankle joint movement during gait. The attachment of a reflective marker on the second metatarsal head and the US probe to the foot was not believed to have affected ankle joint movement during gait, as there was no significant difference in the ankle joint during gait with and without attachment of US probe (Supplemental Fig. 1). Moreover, we may use a cast system to avoid the probe movement; however, it would likely inhibit foot or ankle joint movement. Therefore, the US/MA system is appropriate for quantitatively measuring first TMT joint mobility.

In conclusion, mobility of the first TMT joint in the sagittal plane during the stance phase was quantified using a synchronized US/MA system. The medial cuneiform is displaced in plantar direction during middle stance phase in healthy females without foot disorders than in males. This US/MA system can be used to investigate the pathogenesis of foot disorders, such as the hallux valgus, in future studies.

## Supplementary Information

Supplementary Figure 1.

## Data Availability

The datasets for this study are available upon request from the corresponding author.
