# Supplementary material for: Quantitative evaluation of the vertical mobility of the first tarsometatarsal joint during stance phase of gait
Source: Sci Rep. 2022 Jun 2;12:9246. doi: 10.1038/s41598-022-13425-5 (PMC9163033; doi:10.1038/s41598-022-13425-5)
Supplement: Supplementary file 1 — Supplementary Figure 1. [file 41598_2022_13425_MOESM1_ESM.docx]

**Supplementary data**

As the attachment of the probe to the foot had the possibility of altering the gait pattern, the ankle angle during the stance phase of gait was compared with and without the probe. Ankle angle during the stance phase of gait was obtained from a motion capture analysis system (VICON MX T20-S: Vicon Motion Systems, UK). A paired t-test was used to compare ankle angle during the stance phase of gait with and without the probe. The results showed that there was no significant difference between the two conditions (Supplemental Figure 1).


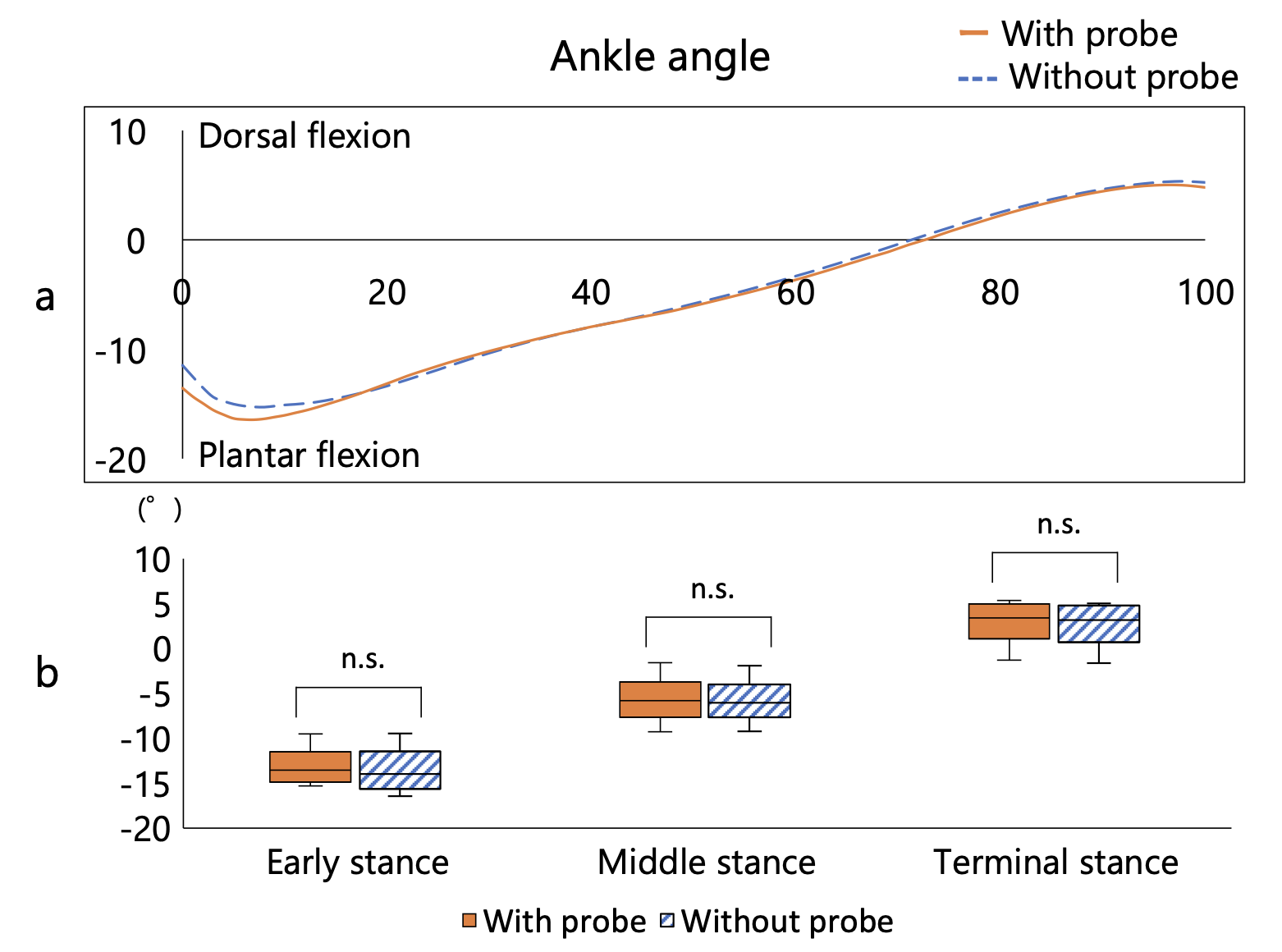


Supplemental Figure 1. Ankle plantar and dorsal flexion angle during one stance phase of gait

Temporal changes in ankle angle in the sagittal plane with and without probe are given (a). Box plots show comparisons of ankle angles during the early, middle, and terminal stance phases (b). n.s.: not significant.
